# EnDovascular therapy plus best medical treatment (BMT) versus BMT alone for medIum distal veSsel occlusion sTroke (DISTAL): An international, multicentre, randomized-controlled, two-arm, assessor-blinded trial

**DOI:** 10.1177/23969873241250212

**Published:** 2024-05-03

**Authors:** Psychogios Marios-Nikos, Brehm Alex, Fiehler Jens, Fragata Isabel, Gralla Jan, Katan Mira, Leker Ronen, Machi Paolo, Ribo Marc, Saver Jeffrey L, Strbian Daniel, van Es Adriaan, Zimmer Claus, Rommers Nikki, Balmer Luzia, Fischer Urs

**Affiliations:** 1 Department of Neuroradiology, University Hospital Basel, Basel, Switzerland; 2 Clinic of Diagnostic and Interventional Neuroradiology, University Hospital Hamburg Eppendorf, Hamburg, Germany; 3 Department of Neuroradiology, Centro Hospitalar Universitário Lisboa Central, Lisboa, Portugal; 4 NOVA Medical School, Universidade NOVA de Lisboa, Lisbon, Portugal; 5 Clinic of Diagnostic and Interventional Neuroradiology, Inselspital Bern, Bern, Switzerland; 6 Clinic of Neurology, University Hospital Basel, Basel, Switzerland; 7 Department of Neurology, Stroke Center, Hadassah Medical Center, Jerusalem, Israel; 8 Clinic of Diagnostic and Interventional Neuroradiology, Hôpitaux universitaires de Genève, Genève, Switzerland; 9 Department of Neurology, Hospital Vall d’Hebron, Barcelona, Spain; 10 Comprehensive Stroke Center and Department of Neurology, David Geffen School of Medicine of UCLA, Los Angeles, CA, USA; 11 Division of Emergency Neurology and Neurocritical care, HUS, Helsinki, Finland; 12 Department of Radiology and Nuclear Medicine, Leiden University Medical Center, Leiden, The Netherlands; 13 Clinic of Diagnostic and Interventional Neuroradiology, Technical University of Munich, Munich, Germany; 14 Department of Clinical Research, University Hospital Basel, Basel, Switzerland; 15 Clinic of Neurology, Inselspital Bern, Bern, Switzerland

**Keywords:** Ischemic stroke, medium vessel occlusion, distal vessel occlusion, endovascular therapy, mechanical thrombectomy

## Abstract

**Rationale:**

Whether endovascular therapy (EVT) in addition to best medical treatment (BMT) in people with acute ischemic stroke (AIS) due to a medium distal vessel occlusion (MDVO) is beneficial remains unclear.

**Aim:**

To determine if people experiencing an AIS due to an isolated MDVO (defined as the co- or non-dominant M2 segment, the M3 or M4 segment of the middle cerebral artery, the A1, A2, or A3 segment of the anterior cerebral artery or the P1, P2 or P3 segment of the posterior cerebral artery) will have superior outcome if treated with EVT in addition to BMT compared to BMT alone.

**Sample size:**

To randomize 526 participants 1:1 to EVT plus BMT or BMT alone.

**Methods and design:**

A multicentre, international, prospective, randomized, open-label, blinded-endpoint (PROBE) superiority trial.

**Outcomes:**

The primary efficacy endpoint is the distribution of disability levels on the modified Rankin Scale at 90 days. Secondary clinical efficacy outcomes include normalized change in National Institutes of Health Stroke Scale score from baseline to day 1, cognitive outcome at 90 days, and health-related quality of life at 90 days. Safety outcomes include all serious adverse events, symptomatic intracranial hemorrhage within 24 h, and all-cause mortality up to 90 days. Secondary imaging outcomes include successful reperfusion at end of EVT procedure and recanalization of target artery at 24 h.

**Discussion:**

DISTAL will inform physicians whether EVT in addition to BMT in people with AIS due to a MDVO is more efficacious than BMT alone.

## Graphical abstract

**Graphical abstract:**
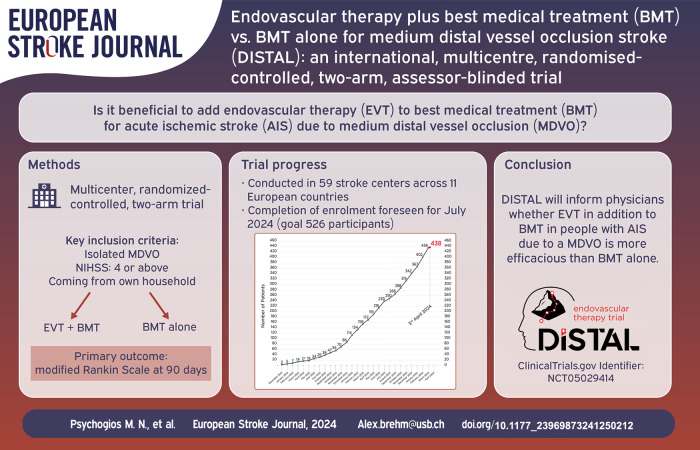

## Introduction

Endovascular therapy (EVT) is beneficial in people with an acute ischemic stroke (AIS) due to large vessel occlusions of the internal carotid artery (ICA), the M1 segment of the middle cerebral artery (MCA) and the basilar artery (BA).^1–3^ Randomized trial evidence is also suggestive that EVT is beneficial for people with an acute occlusion of the dominant M2 segment of the MCA – “M1-like” segments of the M2. An individual participant data meta-analysis of the highly effective reperfusion evaluated in multiple endovascular stroke trials (HERMES) collaboration has indicated a beneficial effect of EVT in AIS people with an occlusion of the M2 segment among whom 123 out of 130 people had a proximal/dominant M2 occlusion (Odds ratio for modified Rankin Scale [mRS] of 2 in favor of EVT 2.68; 95%-Confidence Interval (CI) 1.04–4.81).^4^

Whether people with a medium distal vessel occlusion (MDVO), which is defined as an occlusion of the co- or non-dominant M2, M3 or M4 segment of the MCA, the A1, A2, or A3 segment of the anterior cerebral artery (ACA) or the P1, P2, or P3 segment of the posterior cerebral artery (PCA) should undergo EVT is unclear. Current European Stroke Organisation (ESO) and American Heart Association/American Stroke Association (AHA/ASA) guidelines do not give clear recommendations for or against EVT in people with MDVOs given the lack of randomized controlled trials (RCTs).^5,6^ Despite this lack of evidence, EVT is increasingly offered to people with MDVOs in clinical practice due to increased experience of the interventionalist and improved devices.^7–14^ Outcome in people with MDVOs with best medical therapy (BMT) is insufficient since only 50% achieve excellent functional outcome (mRS 0–1) at 90 days and over 30% do not regain functional independence (mRS 0–2). The high burden of MDVOs and its frequent presentation (approximately 20%–50% of all people with AIS with a visible vessel occlusion in imaging series and 25%–40% in deductive epidemiologic analysis)^15–17^ highlights the importance of new evidence-based treatment approaches.

The DISTAL trial aims to determine whether people with an AIS due to an isolated MDVO will have superior outcome if treated with EVT in addition to BMT compared to BMT alone.

## Methods

### Study design

**Figure 1.**
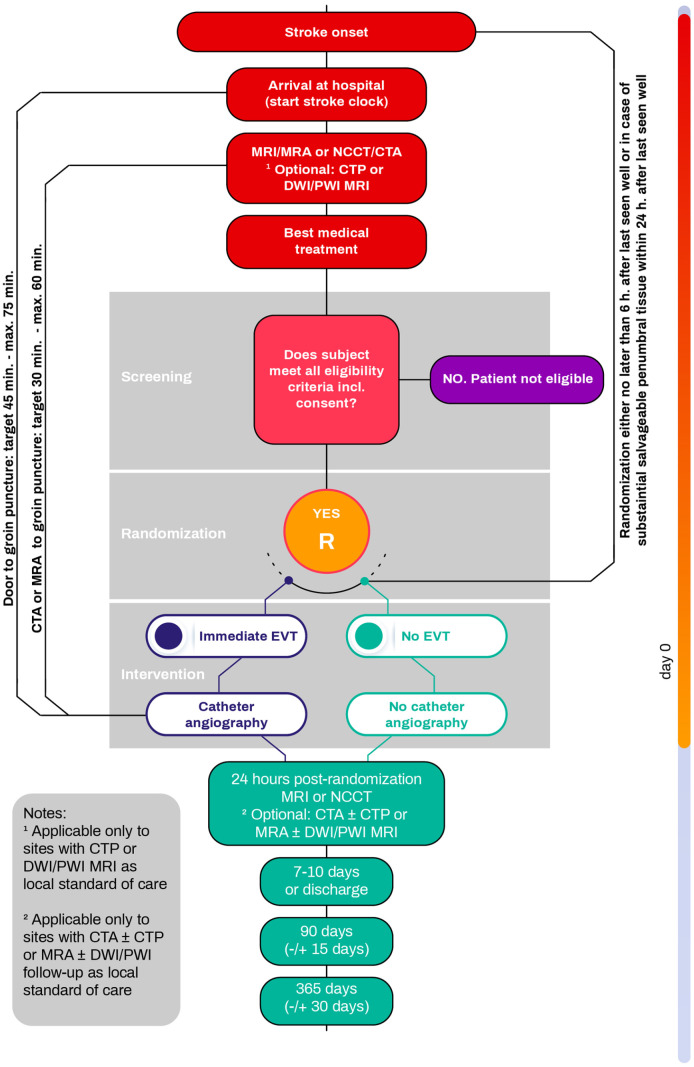
Patient flow chart.

DISTAL is an investigator-initiated, multicentre, international, prospective, randomized (1:1), open-label, blinded-endpoint (PROBE) superiority study. The treatment arm under investigation is EVT plus BMT, the standard arm is BMT alone. Participants flow is depicted in Figure 1. The trial is being conducted in over 50 stroke centers in Switzerland, Belgium, Finland, Germany, Israel, Italy, Portugal, Spain, Sweden, the Netherlands, and the United Kingdom. The first patient was enrolled in December 2021.

### Population

The trial population consist of people who present with an AIS due to an imaging confirmed isolated MDVO. The definition of the co-/or non-dominant M2 segment is based on the perfused territory. An M2 segment is deemed to be co-/or non-dominant if it perfuses less than half of the M1 territory (i.e. less than 100 cc of brain parenchyma on perfusion imaging) or if the occluded M2 segment is clearly smaller than the non-occluded M2 on angiography. The in- and exclusion criteria are listed in Table 1.

**Table 1.** In- and exclusion criteria.

**Inclusion criteria:**
1. Acute ischemic stroke
2. Treatment (arterial puncture) can be initiated 2.1. Within 6 h of last seen well (LSW) **OR** 2.2. Within 6–24 h of LSW **AND** **CT Criteria:** Evidence of a hypoperfusion-hypodensity mismatch (Absence of hypodensity on the non-contrast CT within ⩾90% of the area of the hypoperfused lesion on perfusion CT) **MRI Criteria:** Evidence of a diffusion-hyperintensity mismatch (Absence of hyperintensity on fluid-attenuated inversion recovery (FLAIR) imaging within ⩾90% of the area of the diffusion weighted imaging (DWI) lesion)
3. Isolated medium distal vessel occlusion (i.e. an occlusion of the co-/non-dominant M2, the M3/M4 segment of the MCA, the A1/A2/A3 segment of the ACA or the P1/P2/P3 segment of the PCA) confirmed by CT or MRI Angiography
4. National Institute of Health Stroke Scale Score of ⩾4 points or symptoms deemed clearly disabling by treating physician
5. Age ⩾ 18 years
6. Informed Consent as documented by signature or fulfilling the criteria for emergency consent/ deferral consent
7. Agreement of treating physician to perform endovascular procedure
**Exclusion criteria**
1. Acute intracranial hemorrhage
2. People bedridden or presenting from a nursing home
3. In-Hospital Stroke
4. Known (serious) sensitivity to radiographic contrast agents, nickel, titanium metals or their alloys
5. Foreseeable difficulties in follow-up due to geographic reasons
6. Evidence of an ongoing pregnancy prior to randomization. A negative pregnancy test before randomization is required for all women with child-bearing potential.
7. Known history of arterial tortuosity, pre-existing stent, other arterial disease and/or known disease at the arterial access site that would prevent the device from reaching the target vessel and/or preclude safe recovery after EVT
8. Known, severe comorbidities, which will likely prevent improvement or follow-up (active cancer, alcohol/drug abuse or dementia)
9. Radiological confirmed evidence of mass effect or intracranial tumor (except small meningioma)
10. Radiological confirmed evidence of cerebral vasculitis
11. Evidence of vessel recanalization prior to randomization
12. Participation in another interventional trial

ACA: anterior cerebral artery; CT: computer tomography; EVT: endovascular treatment; MCA: middle cerebral artery; MRI: magnet resonance imaging; PCA: posterior cerebral artery.

### Randomization and blinding

Randomization must be performed within 24 h of last seen well. Participants are randomly assigned to one of the two treatment arms after imaging confirmed an isolated MDVO using probabilistic minimization implemented in a web-based data management system. Allocation is stratified by the National Institutes of Health Stroke Scale (NIHSS). For each participant withdrawing consent before the final-outcome assessment, an additional participant is included. The allocation of a participant is displayed to the treating physicians after randomization. Assessment of the primary outcome at 90 days is performed by an independent and blinded certified rater using validated standards during either a clinical visit or a telephone interview. Raters are certified for the mRS. An independent central core lab evaluates clinical imaging data.

### Treatment

The experimental intervention is EVT. All decisions regarding the performance of EVT are made by the treating physician. The treating physician decides based on local standards and experience on the EVT technique and on the devices and/or medications used for EVT. Administration of intra-arterial thrombolytics, the use of rescue device (e.g. permanent intracranial stents or balloon catheters), and the number of passes (or the decision to stop the intervention due to perceived futility, complications, or safety concerns) is at the discretion of the treating physician.

The control intervention is no EVT. BMT (this includes i.v. thrombolytics) is given in both treatment arms according to local standard of care and/or respective international guidelines (ESO, AHA/ASA). The decision to use any medication including i.v. thrombolytics should be made independent of trial participation. Group allocation should not influence BMT or aftercare. Sites are encouraged to strive to reduce any treatment delays to an absolute minimum. The target for imaging to arterial puncture time is 30–60 min.

### Clinical and imaging evaluation

After clinical evaluation, all participants undergo either non-contrast computed tomography (NCCT) and CT-angiography (CTA) or magnetic resonance imagining (MRI) and MR-angiography (MRA). Perfusion imaging (either with CT or MR) is optional. Clinical examination and radiological follow-up with either NCCT or MRI are performed at 24 ± 6 h. Sites are asked to also perform CTA or MRA, however only NCCT or MRI is mandatory according to the protocol. Follow-up clinical examination is done at 7–10 days or at discharge, if earlier. At 90 ± 14 days, mRS, cognitive status (Montreal Cognitive Assessment), NIHSS and EuroQoL 5D-5L are assessed, whenever possible during a clinical examination or telephone interview. Sites are asked to perform a clinical examination whenever possible. In case it is done over a telephone interview the NIHSS will not be obtained. At 365 ± 30 days mRS, EuroQoL 5-D-5L, and living situation of participants are assessed by a telephone call. All trial procedures are summarized in Table 2.

**Table 2.** Trial schedule.

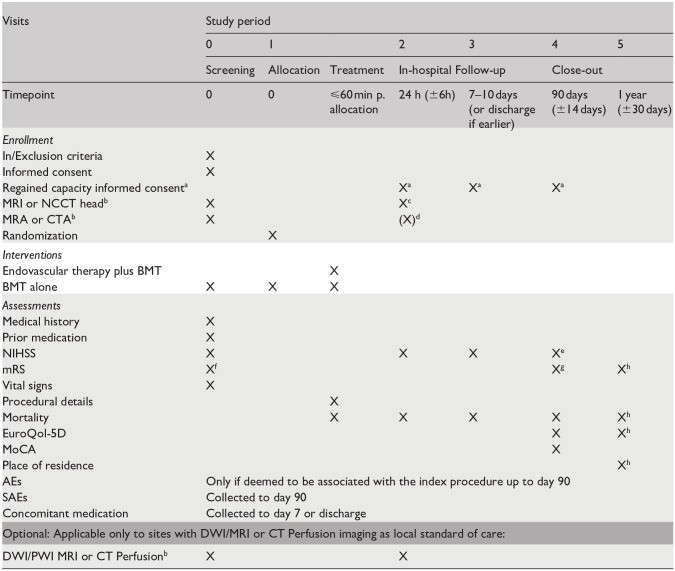							

AE: adverse event; DWI: diffusion weighted imaging; NIHSS: National Institute of Health Score Scale; MoCA: Montreal Cognitive Assessment; mRS: modified Rankin Scale; MRI: magnet resonance imaging; NCCT: non contrast computed tomography; PWI: perfusion weighted imaging; SAE: serious adverse event.

a Post-hoc consent if patient was not able to give consent at trial inclusion (according to national and applicable law).

b Routine Examination for AIS patients.

c MRI Head is preferable but can be substituted by NCCT head if MRI is unavailable or contraindicated.

d Recommended if local standard of care or if an extracranial stent was placed.

e Obtaining the NIHSS also at the visit at day 90 is optional and can be done according to local standard of care.

f Historical (pre-Stroke) mRS can be collected at any time. Pre-stroke mRS is completed by obtaining verification from an individual aware of the subject’s functional status prior to stroke (e.g. family member, friend, etc.).

g At day 90 it is preferred that patients will return to the clinic. If a in clinic visit is not possible due to the patients’ condition, the patient can be contacted by telemedicine or by telephone. Assessment to be performed by an independent evaluator blinded to treatment assignment.

h All endpoints at 1 year will be obtained via telephone call either with (1) the patient, (2) relevant next of kin or (3) family physician.

### Primary outcome

The primary outcome is dependency and disability in daily life actives (measured with the mRS) at 90 ± 14 days after randomization.

### Secondary outcomes

The secondary clinical efficacy outcomes at 24 h are: change in neurologic deficit severity; at 90 days: excellent functional outcome (mRS 0–1), cognitive function and health-related quality of life; and at 1 year: level of dependency and disability in daily life, health-related quality of life and residential status. Safety outcomes are (1) all serious adverse events up to 90-day follow up visit; (2) symptomatic intracranial hemorrhage according to the modified SITS-MOST criteria* within 24 h (±6 h) after randomization; and (3) all-cause mortality within 90 and 365 days after randomization. Technical and imaging efficacy outcomes are (1) Successful reperfusion at the end of EVT procedure (defined as eTICI 2b50–3); and (2) recanalization of target artery at 24 h (±6 h) after randomization, defined as Arterial Occlusion Lesion scale score 2–3 on CTA or MRA.

*Modified SITS-MOST criteria: Symptomatic intracerebral hemorrhage is defined as local or remote parenchymal hemorrhage type 2, subarachnoid hemorrhage, and/or intraventricular hemorrhage on the 24 h (±6 h) post-treatment imaging scan, combined with a neurological deterioration of 4 points or more on the NIHSS from baseline, or from the lowest NIHSS value between baseline and 24 h, or leading to death. The imaging component of the definition will be evaluated by the imaging core lab.

### Sample size calculation

The sample size was calculated by a simulation approach (10,000 times). The sample size calculation is based on the disability outcome distribution of MDVO patients treated with BMT only observed in the INTERSECT and the PRoveIT register and an assumed treatment effect of EVT of 20% relative improvement on the mRS scale.^18^ The mRS distribution was then calculated using this treatment effect. The hypothesis was tested with a univariable proportional odds model for ordinal regression (shift analysis) with treatment group as the only fixed effect. To achieve a power of 80% at an alpha-level of 0.05, a total sample size of 502 participants is required, and with an assumed dropout of 5%, a total of 526 participants will be recruited.

### Statistical analysis

The primary endpoint (distribution of the mRS at 90 days with scores 5 and 6 combined) will be assessed for superiority of EVT plus BMT compared to BMT alone using a proportional odds model. The fixed effect of main interest will be treatment (EVT plus BMT vs BMT alone). Covariates that will be added to the model are age, NIHSS score, pre-stroke mRS, occlusion location, treatment within 6 or between 6 and 24 h, and treatment with intravenous lysis (yes vs no). The treatment effect will be estimated by the adjusted common odds ratio (i.e. likelihood for improvement across all categories of the mRS) with the corresponding 95%-CI. Frequency distributions of mRS will be reported for each treatment arm with the corresponding 95%-CI. If the proportional odds assumption is violated and bidirectional treatment effects are observed, the utility weighted mRS approach will be used.

All secondary clinical efficacy outcomes will be analyzed in a predefined order with the serial gatekeeping strategy for multiplicity control, given that the primary endpoint has reached statistical significance.^19^ The predefined order of testing is: (1) Excellent functional outcome (mRS 0–1 vs mRS 2–6); (2) Normalized change in NIHSS; (3) Health-related quality of life (Euro-Qol 5d); (4) Cognitive function. The binary outcomes will be analyzed using logistic regression models, ordinal outcomes using proportional odds models, and continuous outcomes using linear regression models. All models will be adjusted for the same covariates as the primary analysis. After the serial gatekeeping has failed, we will analyze the subsequent endpoints in an exploratory manner. This means that we will report the appropriate effect estimates with confidence intervals and *p*-values, without the intention of making formal, confirmatory claims about these endpoints.

The primary analysis will be performed according to the intention-to-treat principle on the imputed full analysis set. Missing data will be imputed using multiple imputation by chained equations (MICE), under the assumption that data are missing at random,^20^ for which all available baseline variables will be utilized. As a supplementary analysis, a per protocol analysis for the primary endpoint will be performed, excluding all patients who did not receive treatment as predefined in the study protocol, or had other predefined major protocol deviations. Furthermore, a sensitivity analysis will be performed on the complete case data set excluding cases with imputed data. Safety outcomes will be described in both groups in all randomized patients who received one of the study interventions according to the treatment received by the patient.

All analyses will be performed in R version 4.3.2 or higher. Subgroup analyses will be predefined in the statistical analysis plan that will be finalized and uploaded to a public repository before database closure.

### Data safety monitoring board (DSMB)

An independent DSMB monitors the trial and meets after 150 and 262 of the participants have completed the primary endpoint to review safety data. All relevant safety variables will be described, and treatment groups will be blinded. One prespecified efficacy interim analysis is performed after 262 participants have reached the primary efficacy endpoint. Results of interim analysis will only be presented to the DSMB. The DSMB will consider recommending stopping the trial for efficacy if the Haybittle–Peto boundary (*p* < 0.0001) is crossed for the fixed effect of treatment arm in the adjusted primary analysis. Stopping the trial for futility will be advised if given the observed data, the conditional power of rejecting the null hypothesis (compared with the alternative hypothesis) based on the adjusted primary analysis is less than 10%.

### Study organization and funding

DISTAL is an investigator initiated clinical trial. It is sponsored by the University Hospital Basel and the Principal Investigator and CO-Principal Investigator of the study are Prof. Marios-Nikos Psychogios (Department of Neuroradiology, University Hospital Basel, Basel, Switzerland) and Prof. Urs Fischer (Department of Neurology, University Hospital Basel, Basel, Switzerland). The trial is supported by public grants from the Swiss National Science Foundation (SNSF grant number 33IC30_198783) and the Gottfried und Julia Bangerter-Rhyner-Stiftung (Basel, Switzerland) as well as through unrestricted grants from Stryker Neurovascular Inc., Medtronic Inc., Phenox GmbH, Rapid Medical Inc., and Penumbra Inc. The funders had no role in the design, site selection, planning or conduct of the trial and they will have no role in the analysis of the trial data, the writing of the manuscript or the interpretation of the trial data. The trial is managed by the Department of Neuroradiology, University Hospital Basel, Switzerland. The database, monitoring and statistical analysis are performed by the Department of Clinical Research, University Hospital Basel, Switzerland.

### Trial status

Recruitment is ongoing and 438 patients have been randomized. Completion of enrollment is foreseen in July 2024.

## Discussion

DISTAL will address the question whether EVT in addition to BMT improves the outcome of people with AIS due to MDVOs. It is one of six ongoing trials investigating the effect of EVT on the outcome of people with a MDVO. DISTAL differs from the other trials by its pragmatic approach which allows the physician to choose the device and by its liberal inclusion criteria reflecting everyday clinical routine. The definition of a minimal clinical deficit is an NIHSS of 4 or above or symptoms clearly disabling as judged by the treating physician. This definition was chosen to be able to include people with posterior circulation stroke as their deficits are often not well captured with the NIHSS, due to its focus on motor symptoms. Furthermore, people with pre-morbid deficits but able to live independently at home can also be included in the trial. This approach increases the generalizability of the results to routine clinical practice. From a participants’ perspective, preserving the capacity to live at home is of high importance and should be a priority treatment goal. As most of the MDVO trials utilize similar inclusion criteria and outcome parameters pooling of the data will be possible in the future to analyze subpopulations. An overview of the ongoing trials is given in Table 3.

**Table 3.** Overview of ongoing trials.

Study name	Primary outcome	Planned sample size	Included vessels	Allowed devices	Time-window	Clinical deficit	Imaging based criteria
DISCOUNT (NCT05030142)	mRS (0 – 2)	488 participants	Distal M2 (above mid-height of insula), M3, A1, A2, A3 and P1, P2. P3	Trevo NXT ProVue Retriever, Catchview mini, pReset Lite, Tigertriever 13	Up to 24 h	NIHSS ⩾ 5	None in the early time window. Within the 24 h since last known well, if FLAIR is negative.
DISTAL (NCT05029414)	mRS (shift)	526 participants	Co- or non-dominant M2, M3, M4, A1, A2, A3 and P1, P2. P3	All (open label)	Up to 24 h	NIHSS ⩾ 4 or symptoms clearly disabling	None in the early time window (first 6 h), hypoperfusion – hypodensity mismatch in late window (6–24 h)
DISTALS (NCT05152524)	Successful reperfusion without symptomatic intracranial hemorrhage	168 participants	Distal vessel with vessel diameter ⩾ 1.5 mm	Rapid Medical Tigertriever 13	Up to 24 h	Disabling deficits in territory of distal occlusion	Ischemic core less than 50% of perfusion lesion volume
DUSK (NCT05983757)	mRS (shift)	564 participants	Non-dominant M2, M3, A1, A2, A3 and P1, P2, P3	Trevo NXT ProVue Retrievers, AXS Vecta 46 Intermediate Catheter, AXS Catalyst 5 Distal Access Catheter	Up to 12 h	NIHSS > 5 or NIHSS 3–5 with disabling deficit	Target mismatch: Ischemic core < 50 cc, Mismatch volume > 10 cc, Mismatch ratio > 1.4
ESCAPE-MeVO (NCT05151172)	mRS (shift analysis)	530 participants	M2, M3, A2, A3 and P2, P3	Medtronic Solitaire X 3 mm	Up to 12 hours	NIHSS > 5 or NIHSS 3–5 with disabling deficit	ASPECTS ⩾ 8
FRONTIERS-AP (ACTRN12621001746820p)	mRS (0–1 or no change)	240 participants	M2, M3, A1 and A2	Stryker Trevo XP ProVue 3 × 20 mm	Up to 9 h	NIHSS ⩾ 5 or dysphasia	Hypodensity in less than 50% of perfused territory

mRS: modified Rankin Scale; NIHSS: National Institute of Health Stroke Scale; ASPECTS: Alberta Stroke Program Early CT Score.

A potential challenge inherent to all MDVO trials is that physicians might have already to a certain degree “decided” which therapy is the best, leading to a potential bias due to systematically excluding potential people based on severity of symptoms or other factors such as age or premorbid status. Similar effects were observed in previous EVT trials examining the effect of EVT on outcome in basilar artery occlusions.^21,22^ We have actively tried to reduce this bias during the trial by discussing this topic with the sites and promoting the inclusion of all eligible people. Another possible limitation is the comparable low number of ACA and PCA occlusions which might limit the possibility to address efficacy and safety of EVT in these subgroups.

## Conclusion

The DISTAL trial will provide high quality evidence regarding the question whether EVT in addition to BMT is improving the outcome of AIS people due to MDVO.
